# A Rare Case of Multicentric Reticulohistiocytosis with Concurrent Rheumatoid Arthritis

**DOI:** 10.7759/cureus.5476

**Published:** 2019-08-24

**Authors:** Anupama Behera, Sujata Devi, Satyabrata Guru, Madhusmita Sethy

**Affiliations:** 1 Internal Medicine, All India Institute of Medical Sciences, Bhubaneswar, IND; 2 Pathology, All India Institute of Medical Sciences, Bhubaneswar, IND

**Keywords:** rheumatoid arthritis, multicentric reticulohistiocytosis, distal interphalangeal joint, methotrexate

## Abstract

Multicentric reticulohistiocytosis (MRH) is a rare multisystem macrophage disorder of unknown etiology characterized by papulonodular skin and mucosal lesions, rapidly progressive erosive symmetric polyarthritis, and inflammation of internal organs. Most often, it is misdiagnosed as rheumatoid arthritis (RA). Here, we report the case of a 60-year-old woman found to have features of both MRH and RA with positive rheumatoid factor and high titer of anti-cyclic citrullinated peptide antibody in serum. It was confirmed by a histopathology of skin lesions, which showed diffuse histiocytic infiltrate with multinucleated giant cells. She was treated with methotrexate, hydroxychloroquine, corticosteroids, and nonsteroidal anti-inflammatory drugs and bisphosphonate.

## Introduction

Multicentric reticulohistiocytosis (MRH) and rheumatoid arthritis (RA) are destructive arthritic and skin disorders with a very rare association between them. MRH is characterized by erosive polyarthritis and papulonodular lesions over the skin, mucous membranes, and other internal organs [1]. It may progress to arthritis mutilans in 45% of cases [2]. Here, we report a case of RA with concurrent MRH.

## Case presentation

**Figure 1:**
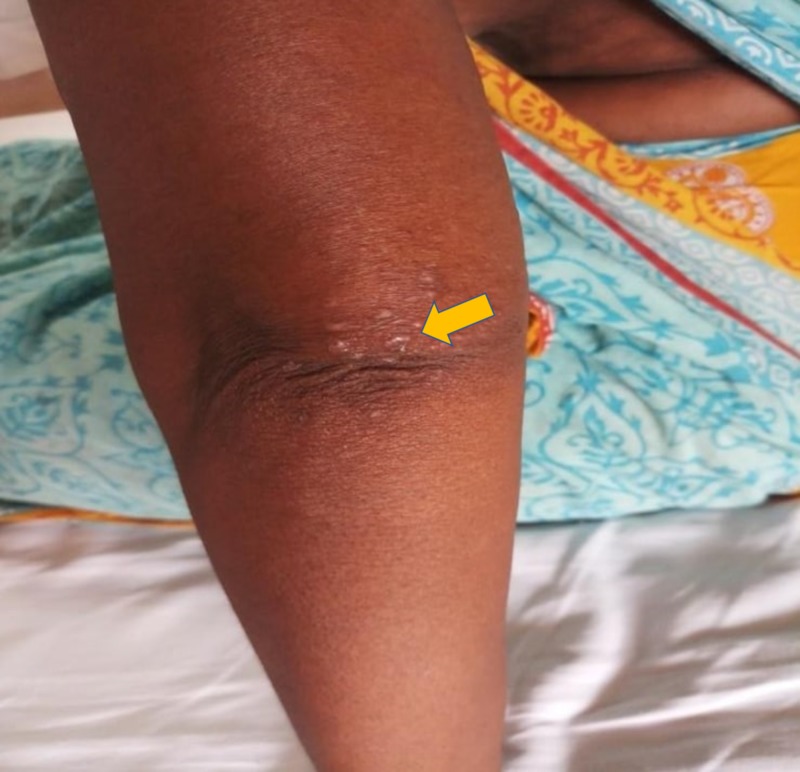
Papulonodular lesion over the left elbow

**Figure 2:**
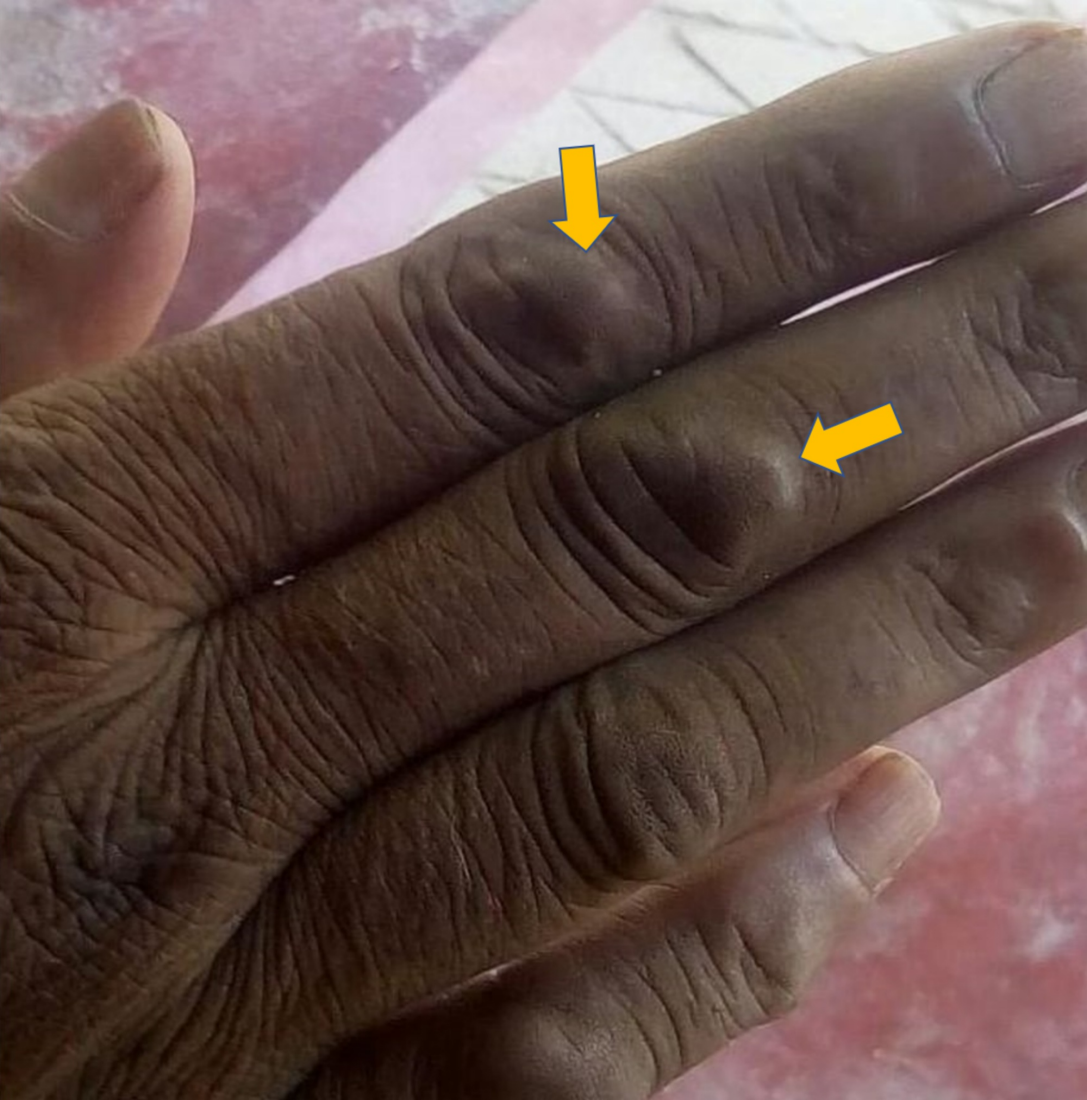
Papulonodular lesion over dorsum of the right hand

**Figure 3:**
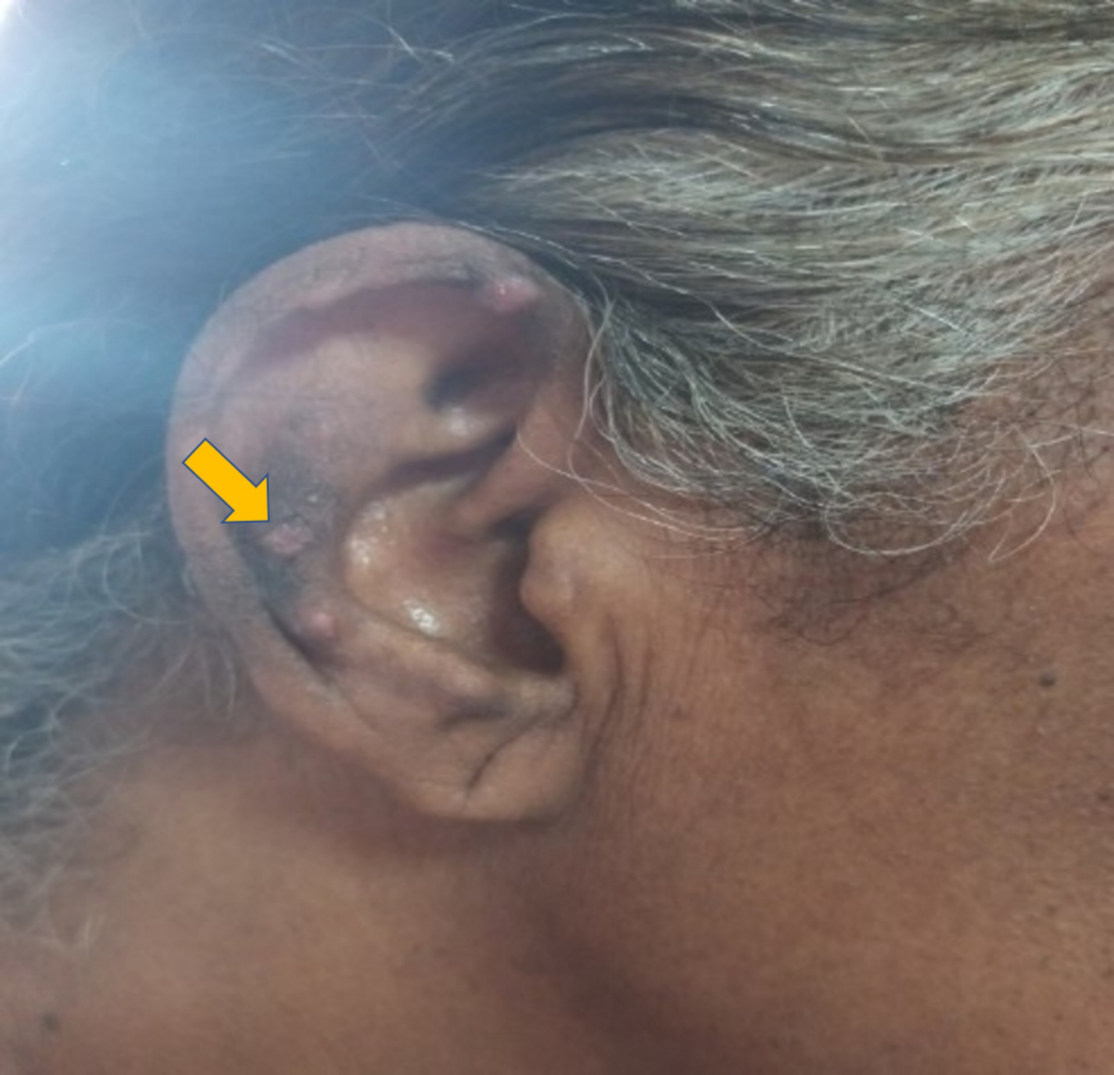
Papulonodular lesion of the right pinnae

A 60-year-old woman presented with concerns of symmetrical polyarthritis occurring for the past 30 years. She was experiencing morning stiffness of more than two hours' duration for which she was taking anti-inflammatory medications. For the past year, she has noticed papulonodular pruritic skin lesions over the scalp, trunk, extensor aspect of the elbow (as seen in Figure 1), dorsum of the hands (as seen in Figure 2), and pinnae (as seen in Figure 3) with a history of spontaneous remission and recurrence. She was confirmed to have diabetes managed by insulin therapy. Upon physical examination, the patient presented with mild pallor and brownish violaceous papulonodular lesions over the pinnae, scalp, elbow, right forearm, and dorsum of the hands and back. A musculoskeletal system examination revealed synovitis of the knee, ankle, shoulder, elbow, wrist, metacarpophalangeal (MCP) joints, proximal interphalangeal (PIP) joints, metatarsophalangeal joints, and sparing distal interphalangeal (DIP) joints without any deformity. Other systems were unremarkable.

**Figure 4:**
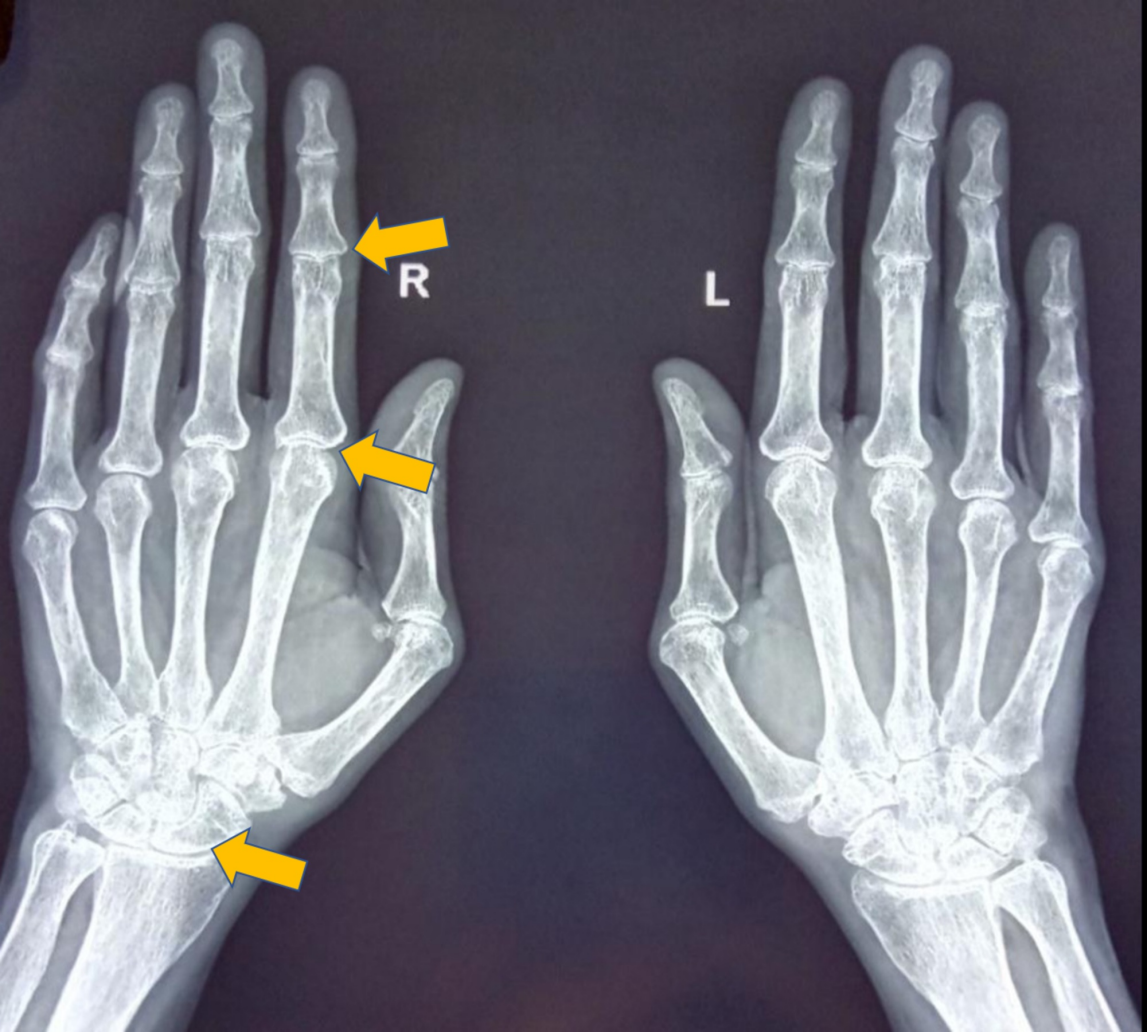
X-ray of both hands revealed subchondral erosion of the wrist, PIP, and MCP joints along with narrowing of these joint spaces with juxtaarticular osteopenia PIP: proximal interphalangeal; MCP: metacarpophalangeal.

**Figure 5:**
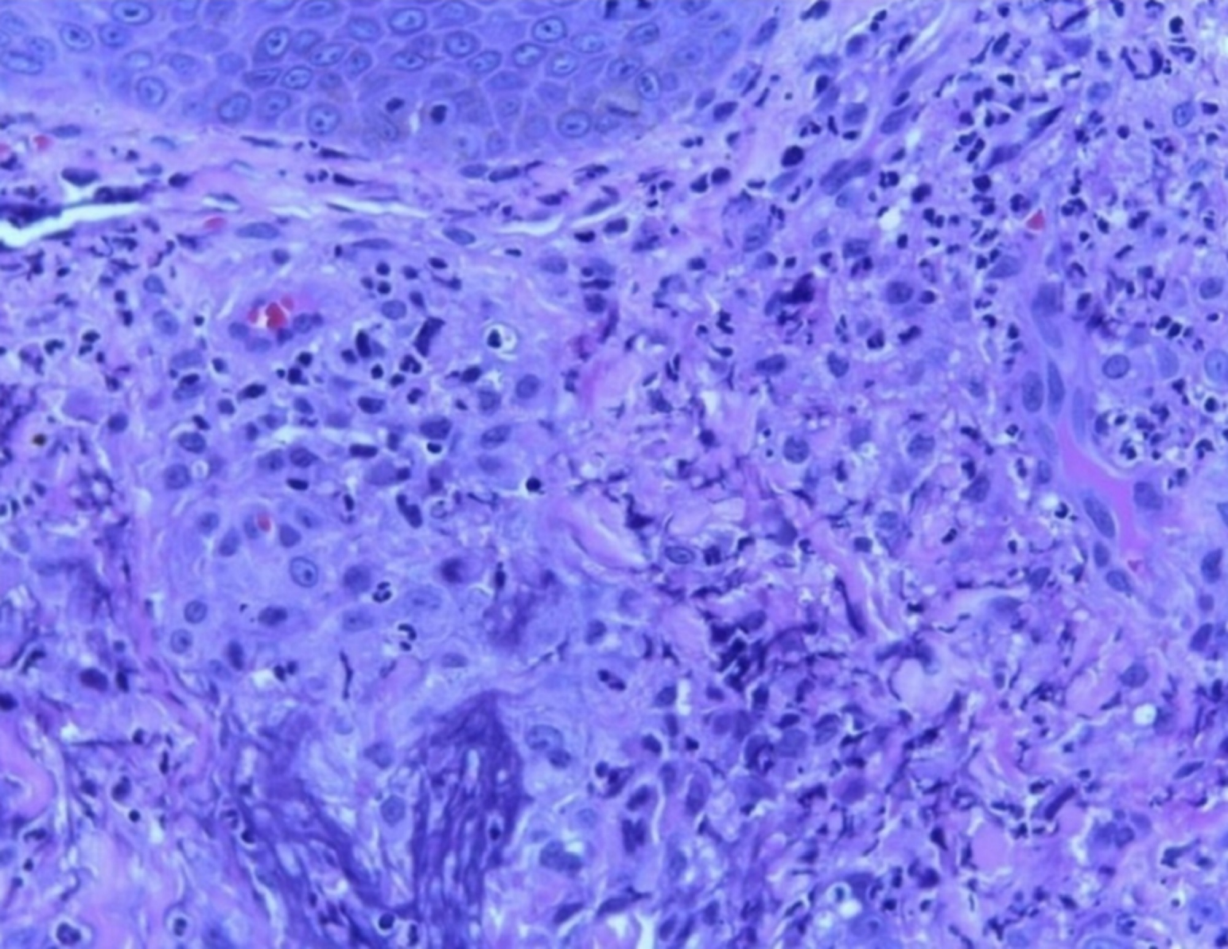
Sheets of histiocytes in the dermis with occasional multinucleated giant cell (hematoxylin and eosin (H&E) stain with 10x magnification)

**Figure 6:**
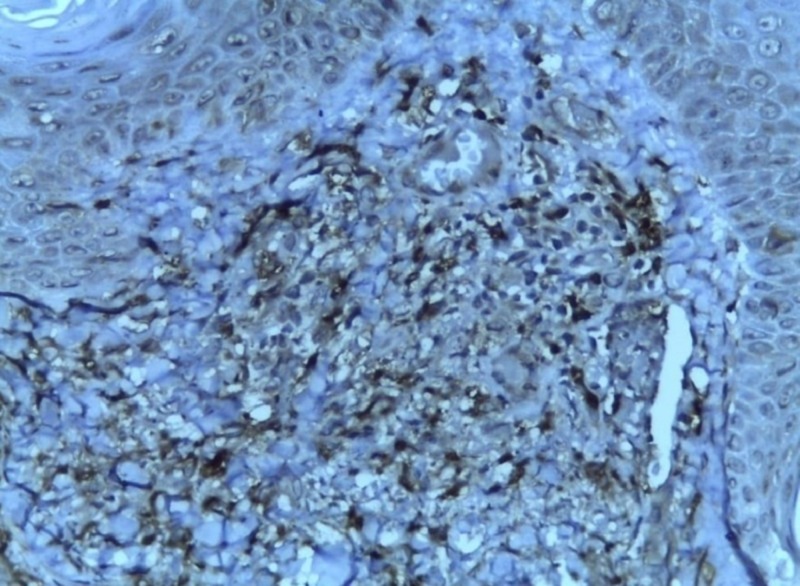
CD163 diffuse positive (immunohistochemistry with 10x magnification)

**Figure 7:**
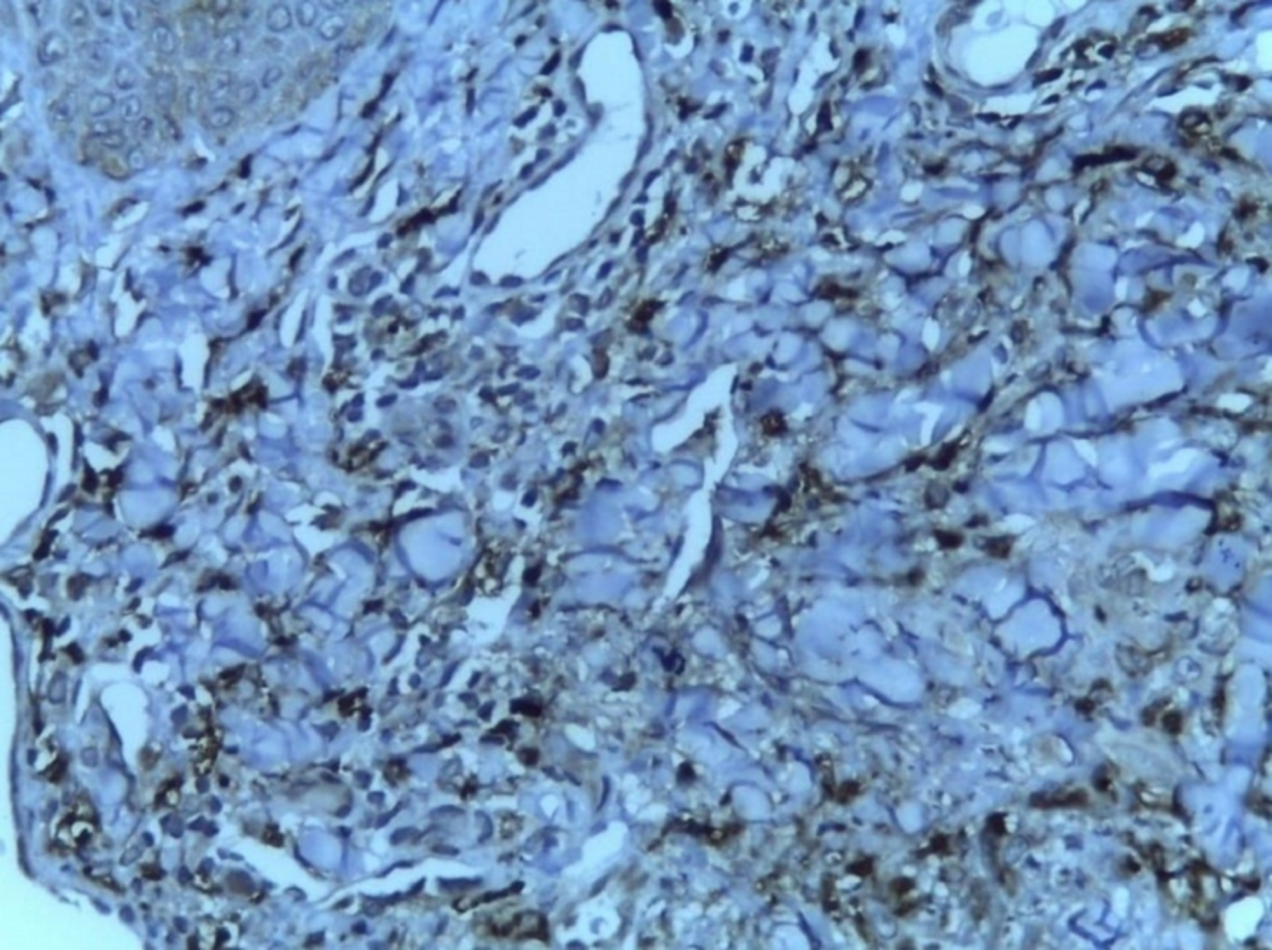
Positive for CD68 (immunohistochemistry with 20x magnification)

**Figure 8:**
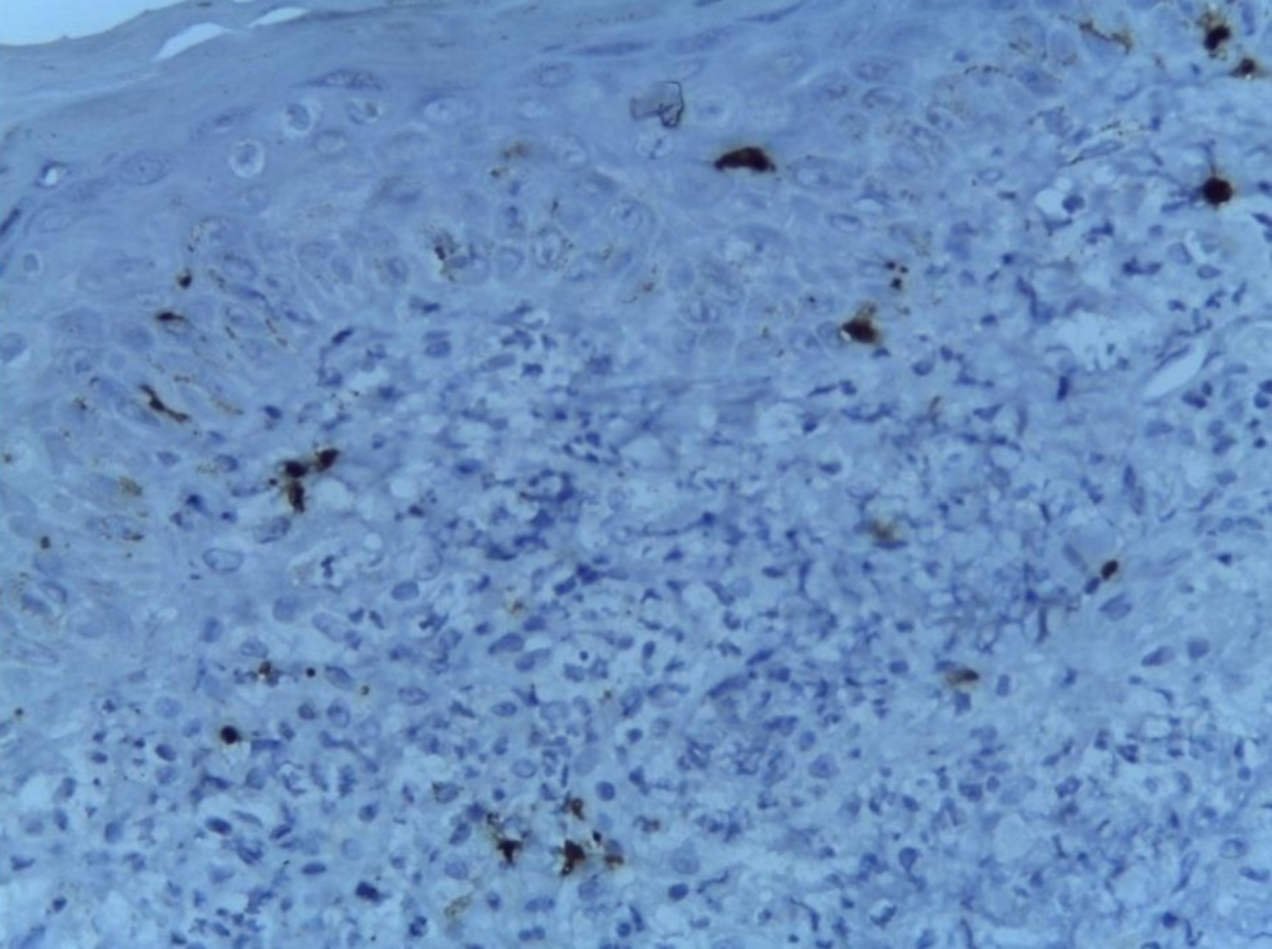
Negative for S100 (immunohistochemistry with 20x magnification)

Laboratory tests revealed microcytic, hypochromic anemia, elevated erythrocyte sedimentation rate (98 mm/first hour), positive rheumatoid factor, and high anti-cyclic citrullinated peptide antibody (CCP) titer of >200 U/mL. C-reactive protein value was 120 mg/L, and serum antinuclear antibody was negative. An X-ray of both hands showed subchondral erosion of wrist, PIP, and MCP joints along with narrowing of these joint spaces with juxta-articular osteopenia (as seen in Figure 4), features consistent with RA. Histopathology of the skin nodule revealed diffuse histiocytic infiltration with occasional multi-nucleated giant cells (as seen in Figure 5), and it was stained positive for CD163 (as seen in Figure 6) and CD68 (as seen in Figure 7) but negatively stained for S100 (Figure 8). She was screened for an associated internal malignancy such as hematological, breast, stomach, or cervix but was found to be negative.

**Figure 9:**
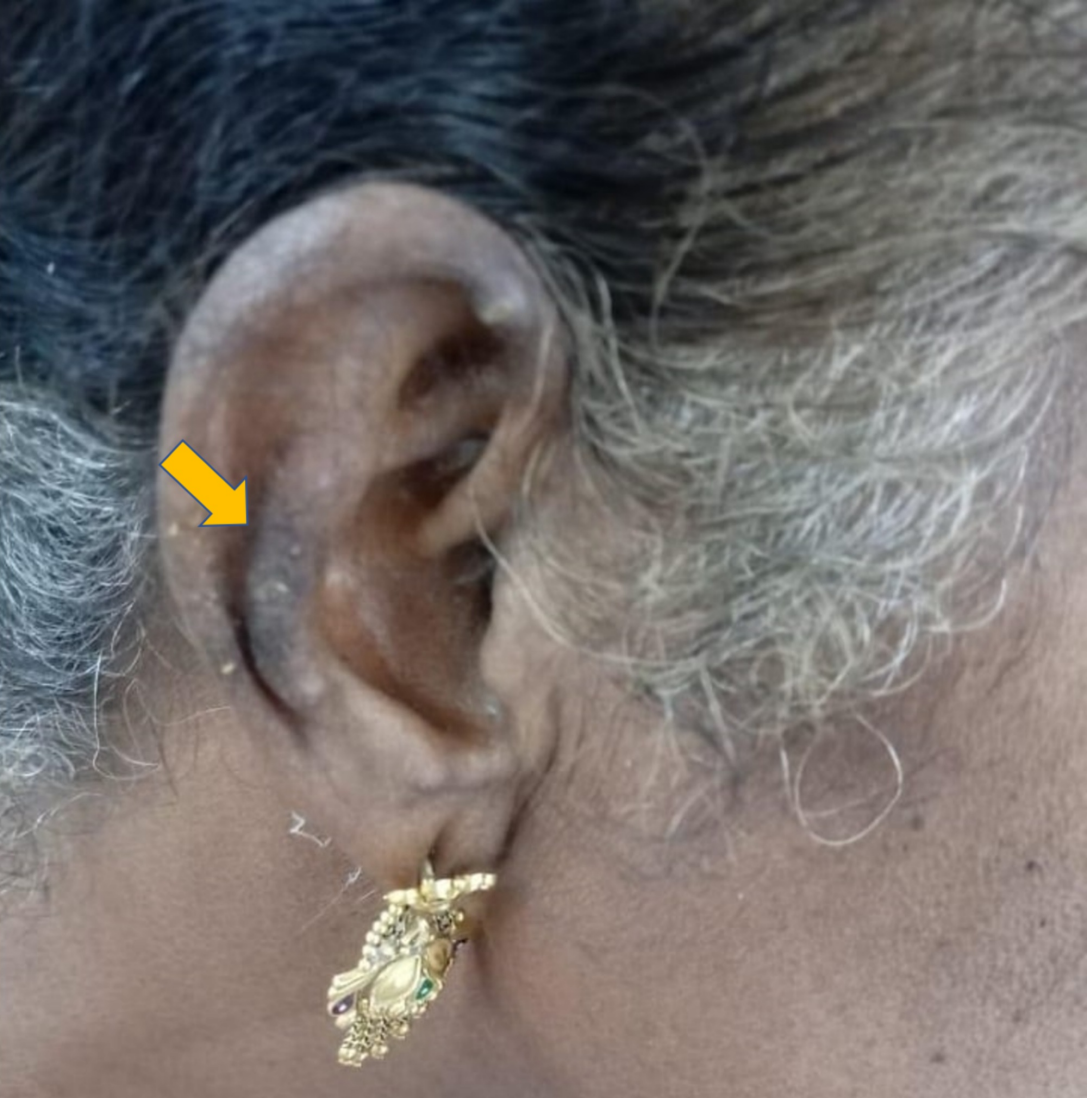
Follow-up after six months: right pinnae

**Figure 10:**
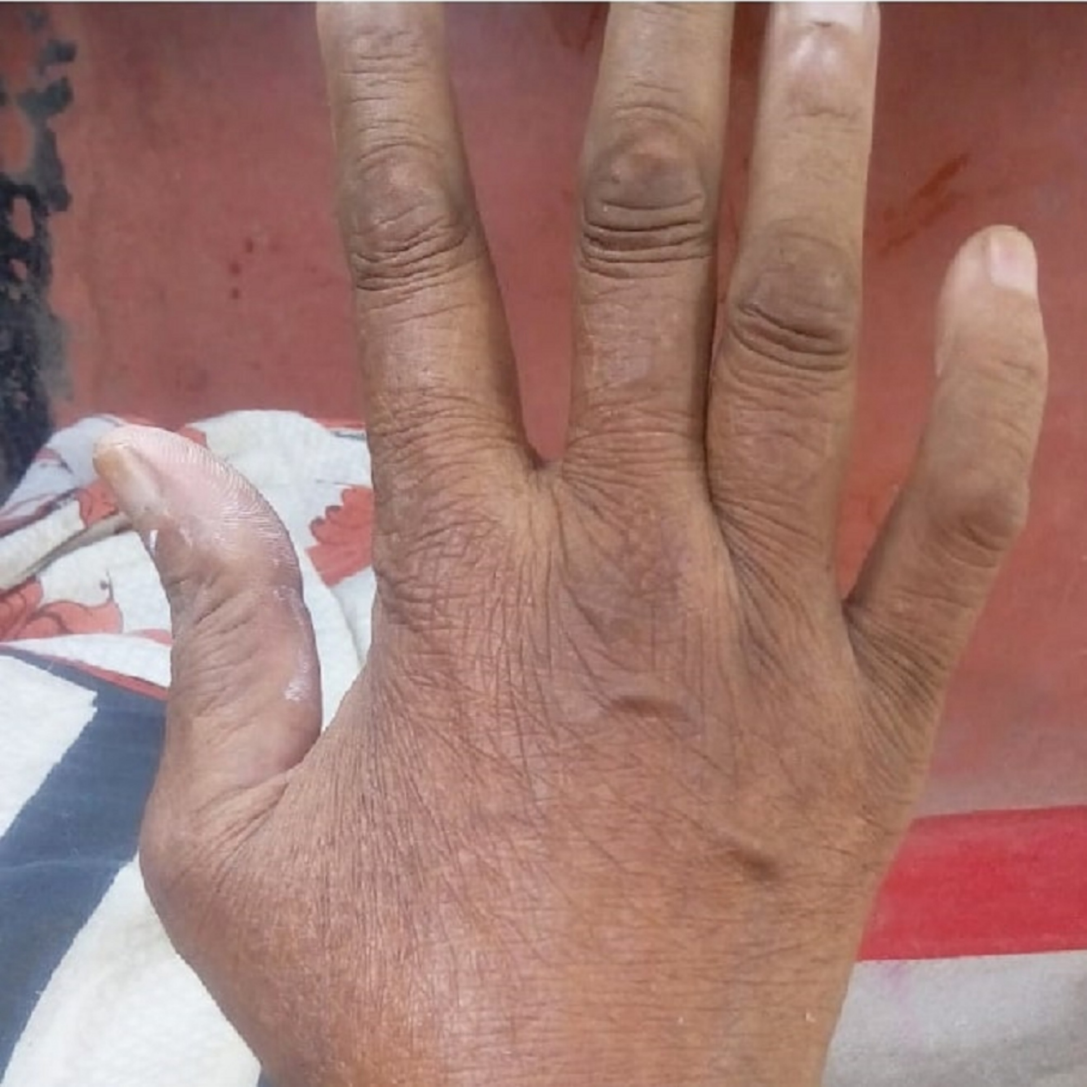
Follow-up after six months: hand

A diagnosis of RA with MRH was made with the combination of clinical pictures, including characteristic skin lesion, confirmatory histopathological finding, X-ray finding without DIP joint involvement, slow progression of arthritis with positive rheumatoid factor, and high anti-CCP antibody titer. She was prescribed methotrexate 10 mg once weekly, prednisolone 10 mg daily, hydroxychloroquine 300 mg daily, and ibandronate 150 mg once monthly for six months. She came for follow-up after six months, at which time her reddish-brown nodules had disappeared, and her synovitis had improved (Figures 9-10).

## Discussion

MRH is a rare disease of unknown etiology with reddish-brown papules and nodules over the skin and nasal and oropharyngeal mucosal surface, associated with a destructive and erosive polyarthritis. Associated complaints such as fever and weight loss are commonly present. MRH with the association of RA is very rare. MRH is usually seen in the fourth decade of life with female preponderance [3-4]. Arthritis in MRH is inflammatory and symmetrical. The most common involvement of the interphalangeal joint of the hand is DIP, accounting for 75% of patients. Symptoms of arthritis may wax and wane or rapidly progress to severe joint destruction, leading to arthritis mutilans; however, in our case, DIP is spared, giving a diagnostic clue of RA distinguishing mainly from psoriatic arthritis and MRH [5]. Skin involvement is a characteristic feature of MRH; it may be the presenting feature, and sometimes the only sign of the disease. It is usually described as reddish-brown papulonodular nonpruritic or pruritic lesions that occur in isolation or clusters or crops with a cobblestone appearance. A thorough investigation should be conducted to exclude internal malignancies such as hematological, breast, stomach, or cervix in patients with MRH [6]. If there are only skin lesions but no arthritis, there is usually no association with cancer. Laboratory investigations should include elevated erythrocyte sedimentation rate, C-reactive protein, anemia, lipid profile, and rheumatoid factor and anti-CCP antibody. Imaging should include X-ray, magnetic resonance imaging of involving joints, skin or synovial tissue biopsy for MRH, which usually demonstrates multinucleated giant cells, mononuclear histiocytes containing periodic acid-Schiff-positive eosinophilic ground glass cytoplasm [3]. As few cases with a combination of MRH and RA are reported, treatment continues to be experimental and includes methotrexate, nonsteroidal anti-inflammatory drugs, hydroxychloroquine, corticosteroid [7], and leflunomide [8]. It has been shown that bisphosphonates such as alendronate and zoledronic acid are an effective treatment for skin nodules as well as arthritis in MRH, suggesting that MRH may be a systemic osteoclastic disease [9].

## Conclusions

Coexistence of MRH with RA is very rare with few cases reported worldwide; therefore, owing to its rarity, diagnosis can be challenging. Hence, clinicians need to have a high index of suspicion if a patient presents with erosive polyarthritis associated with papulonodular skin lesions. A definitive diagnosis of MRH rests upon histological examination of biopsy specimens. Careful analysis of clinical features are the keys to the early diagnosis of this disease.
